# Supplementary material for: Synaptic activity controls local exposure of an 'eat-me' signal via ANO3-ITPR1 signaling
Source: bioRxiv. 2026 Jun 30:2026.06.26.734915. Preprint. [Version 1] doi: 10.64898/2026.06.26.734915 (PMC13344984; doi:10.64898/2026.06.26.734915)
Supplement: Supplement 2 [file NIHPP2026.06.26.734915v1-supplement-2.pdf]

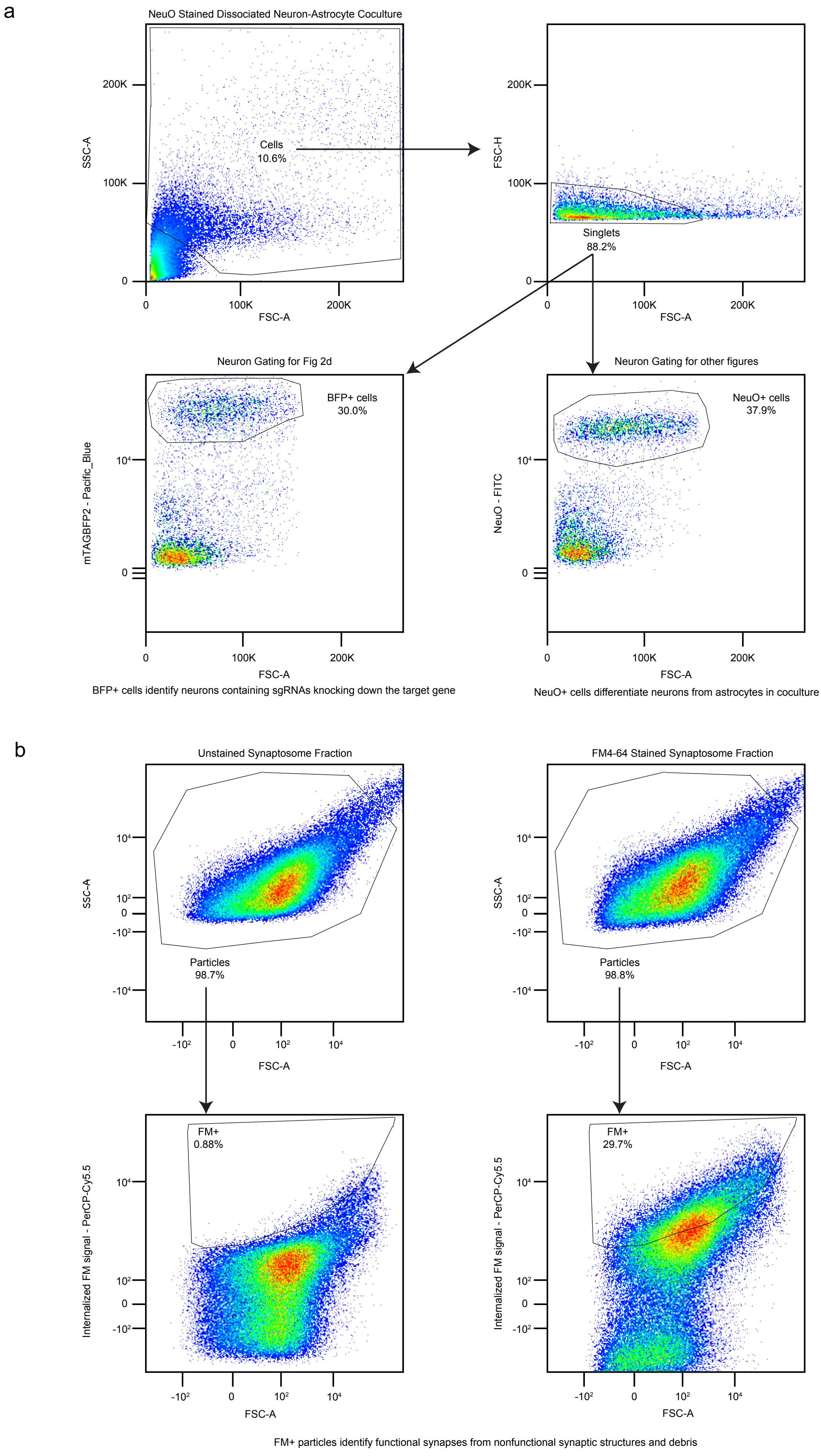

Supplementary Figure 1
Flow cytometry gating strategies. Representative flow cytometry data highlighting (a) somatic flow cytometry gating strategy and (b) synaptosome flow cytometry gating strategy.
